# Investigating eHealth Lifestyle Interventions for Vulnerable Pregnant Women: Scoping Review of Facilitators and Barriers

**DOI:** 10.2196/54366

**Published:** 2024-12-20

**Authors:** Ashley JP Smit, Isra Al-Dhahir, Lieke Schiphof-Godart, Linda D Breeman, Andrea WM Evers, Koen FM Joosten

**Affiliations:** 1 Department of Neonatal and Pediatric Intensive Care Erasmus Medical Center Sophia Children's Hospital Rotterdam Netherlands; 2 Faculty of Social and Behavioral Sciences Health, Medical and Neuropsychology Unit Leiden University Leiden Netherlands; 3 Department of Medical Informatics Erasmus University Medical Center Rotterdam Rotterdam Netherlands; 4 Medical Delta Leiden University, Delft University of Technology, Erasmus University Delft Netherlands

**Keywords:** eHealth, pregnancy, vulnerability, socioeconomic status, lifestyle intervention, intervention development, barriers, facilitators, mobile phone, PRISMA

## Abstract

**Background:**

The maintenance of a healthy lifestyle significantly influences pregnancy outcomes. Certain pregnant women are more at risk of engaging in unhealthy behaviors due to factors such as having a low socioeconomic position and low social capital. eHealth interventions tailored to pregnant women affected by these vulnerability factors can provide support and motivation for healthier choices. However, there is still a lack of insight into how interventions for this target group are best designed, used, and implemented and how vulnerable pregnant women are best reached.

**Objective:**

This review aimed to identify the strategies used in the design, reach, use, and implementation phases of eHealth lifestyle interventions for vulnerable pregnant women; assess whether these strategies acted as facilitators; and identify barriers that were encountered.

**Methods:**

We conducted a search on MEDLINE, Embase, Web of Science, CINAHL, and Google Scholar for studies that described an eHealth intervention for vulnerable pregnant women focusing on at least one lifestyle component (diet, physical activity, alcohol consumption, smoking, stress, or sleep) and provided information on the design, reach, use, or implementation of the intervention.

**Results:**

The literature search identified 3904 records, of which 29 (0.74%) met our inclusion criteria. These 29 articles described 20 eHealth lifestyle interventions, which were primarily delivered through apps and frequently targeted multiple lifestyle components simultaneously. Barriers identified in the design and use phases included financial aspects (eg, budgetary constraints) and technological challenges for the target group (eg, limited internet connectivity). In addition, barriers were encountered in reaching vulnerable pregnant women, including a lack of interest and time constraints among eligible participants and limited support from health care providers. Facilitators identified in the design and use phases included collaborating with the target group and other stakeholders (eg, health care providers), leveraging existing eHealth platforms for modifications or extensions, and adhering to clinical and best practice guidelines and behavior change frameworks. Furthermore, tailoring (eg, matching the content of the intervention to the target groups’ norms and values) and the use of incentives (eg, payments for abstaining from unhealthy behavior) were identified as potential facilitators to eHealth use. Facilitators in the interventions’ reach and implementation phases included stakeholder collaboration and a low workload for the intervention deliverers involved in these phases.

**Conclusions:**

This scoping review offers a comprehensive overview of strategies used in different phases of eHealth lifestyle interventions for vulnerable pregnant women, highlighting specific barriers and facilitators. Limited reporting on the impact of the strategies used and barriers encountered hinders a complete identification of facilitators and barriers. Nevertheless, this review sheds light on how to optimize the development of eHealth lifestyle interventions for vulnerable pregnant women, ultimately enhancing the health of both future mothers and their offspring.

## Introduction

### Background

Maintaining a healthy lifestyle during pregnancy benefits pregnancy outcomes and the health of the developing fetus in particular. For example, the maternal diet plays a significant role in embryonic growth and development [1,2], and engaging in physical activity during pregnancy has been associated with a decreased risk of conditions such as excessive maternal weight gain, preeclampsia, and gestational diabetes mellitus [3]. On the other hand, maternal smoking and alcohol use are associated with an increased risk of preterm birth, among many other detrimental outcomes [4-6]. In addition, high levels of stress during pregnancy have been associated with various adverse outcomes for mother and child, among them preterm birth [7]. However, not all pregnant women maintain healthy lifestyles. Various studies have found that most women in the periconceptional period have inadequate dietary intake, 40% to 78% engage in insufficient physical activity, and 5% to 14% smoke [8,9]. Furthermore, 45% of pregnant women experience stress [8]. These lifestyle behaviors are often negatively affected by nonmedical vulnerability factors such as a low level of education, a low socioeconomic position (SEP), and low social capital [10]. Therefore, pregnant women affected by these vulnerability factors (henceforth referred to as “vulnerable pregnant women”) are more likely to engage in unhealthy behaviors. Acknowledging the intersectionality of these vulnerability factors is crucial as they often compound each other’s effects, enhancing health disparities [11]. For instance, a pregnant woman with a low income may have limited access to nutritious food and simultaneously feel stressed due to financial problems. Similarly, a pregnant woman with a low level of education may encounter barriers in understanding health-related information and accessing appropriate support systems. In addition, cultural beliefs and practices can significantly influence lifestyle behaviors during pregnancy. For instance, certain cultural norms dictate dietary preferences or restrictions during pregnancy, influencing the nutritional intake of pregnant women [12].

The pregnancy period offers a unique opportunity to improve maternal health and, consequently, fetal health, rendering it a crucial time in which vulnerable pregnant women should be encouraged to adopt a healthier lifestyle [13]. Considering that many pregnant women use the internet and smartphone apps as a source of information on pregnancy [14], eHealth interventions targeting the lifestyle of vulnerable pregnant women have the potential to support them in making healthier choices. For example, a tablet delivered lifestyle intervention for underserved pregnant women (those who lack access to essential resources and support during pregnancy, often due to socioeconomic barriers) significantly reduced the number of risk behaviors, particularly in the areas of stress and smoking, and increased fruit and vegetable consumption [15]. The use of digital tools to deliver interventions offers many advantages. First, given the widespread access to the internet among the population [16], eHealth lifestyle interventions are highly accessible. Furthermore, the ability to tailor interventions toward the specific needs and values of their users can increase their effectiveness by enhancing user participation and engagement [17]. These advantages enable these interventions to reach diverse populations. However, despite the many advantages of eHealth lifestyle interventions for vulnerable pregnant women, challenges might be encountered in various phases of their development. For instance, when attempting to recruit participants for a smoking cessation app, researchers encountered difficulties due to a lack of interest among Medicaid-eligible pregnant women [18]. Furthermore, difficulties associated with downloading an app deterred pregnant women from using the eHealth lifestyle intervention Health-e Babies [19]. In addition, despite adapting the content to a level accessible to early-stage readers, a study by Song et al [20] revealed that a third of their participants had difficulty understanding the information provided through SMS text messages. This emphasizes the necessity for developers of eHealth lifestyle interventions targeting vulnerable pregnant women to consider the particular needs and skills of their target group. Research has underscored the significance of eHealth users possessing adequate literacy levels and proficient digital skills [21]. However, it is important to recognize that these prerequisites may present additional challenges for vulnerable groups [22,23].

### This Study

Despite an increasing number of eHealth lifestyle interventions developed for vulnerable pregnant women, there is still a lack of insight into how these interventions are designed, used, and implemented and how vulnerable pregnant women are reached by these interventions. To address this gap, this study sought to extract insights from studies on existing eHealth lifestyle interventions developed for vulnerable pregnant women. These studies on developed interventions offer valuable information regarding their components, challenges faced, and strategies used in each phase (design, reach, use, and implementation) and, therefore, can serve as a valuable resource to guide future researchers in the development or adaptation of eHealth lifestyle interventions for vulnerable pregnant women. Therefore, this scoping review aimed to (1) identify the strategies used and barriers encountered in the design, reach, use, and implementation phase of existing eHealth lifestyle interventions for vulnerable pregnant women; and (2) determine whether these strategies acted as facilitators in the aforementioned phases to provide future developers with an overview of the available knowledge regarding the impact of these strategies.

## Methods

### Design: Scoping Review

As the research area of eHealth lifestyle interventions for vulnerable pregnant women is still in its infancy, a scoping review was chosen as the appropriate method to summarize and disseminate research findings, allowing for the inclusion of literature with varying types of methodological designs. We conducted the search for this scoping review in February 2023 and conducted an updated search in June 2023. We did not publish a review protocol. The conduct of the scoping review was guided by the methodological framework for scoping reviews by Arksey and O’Malley [24] and the 2018 PRISMA-ScR (Preferred Reporting Items for Systematic Reviews and Meta-Analyses extension for Scoping Reviews) checklist (Multimedia Appendix 1) [25].

### Search Strategy

An experienced librarian from Erasmus Medical Center formulated a search strategy together with the first author (AJPS) and conducted a literature search addressing the research objectives (Multimedia Appendix 2). The search strategy included key terms for pregnancy, digital interventions, and lifestyle components. Vulnerability was not part of the search strategy. Instead, the titles and abstracts were screened for vulnerability factors. Vulnerability was defined as characteristics that stratify health opportunities and outcomes based on the PROGRESS-Plus framework by Cochrane [26] and can refer to place of residence, race, ethnicity, culture, and language (henceforth referred to as “ethnicity”), educational level, SEP, social capital, and age. We included low health and digital literacy as additional vulnerability factors [27,28]. In addition, the authors of the included articles had to specifically mention the characteristic as contributing to disparities in health opportunities or outcomes. The databases searched included MEDLINE (1946-present), Embase (1971-present), Web of Science (1975-present), CINAHL (1982-present), and Google Scholar. Duplicate findings were removed. Furthermore, the reference lists of relevant reviews and of the included articles after full-text screening were examined to identify additional relevant articles.

### Eligibility Criteria and Screening

Abstract screening was conducted using ASReview (version 1.1). ASReview is a free open-source screening assistant tool that uses machine learning to assist the reviewer in literature screening. When articles are included or excluded by the reviewer within this software, the ASReview algorithm learns which articles are relevant for the reviewer and adjusts the order of the articles to present the most relevant first. In this way, ASReview allows for a more efficient and time-saving manner of screening articles. The predefined stopping rules for screening entailed screening a minimum of 36.1% of the articles and encountering 25 consecutive nonrelevant articles. With these criteria, it was expected that no more relevant articles would be identified among the remaining unscreened articles [29]. The first author (AJPS) screened the articles based on titles and abstracts against the inclusion and exclusion criteria (Textbox 1). To ensure that the interventions included in this scoping review were relevant for populations from high-income countries, we limited the studies to those conducted in high-income countries as defined in the World Economic Situation and Prospects 2023 report by the United Nations [30]. When uncertainty existed about the relevance of the article based on the title and abstract, or when no abstract was available, the article was included for full-text screening as well. After the initial abstract screening, AJPS performed a full-text screening in which articles were assessed for a second time against the inclusion and exclusion criteria. Any uncertainty was discussed with the other authors. The reasons for exclusion of articles after full-text screening were recorded.

**Inclusion and exclusion criteria**

**Inclusion criteria**

- Description of an eHealth intervention that aims to change modifiable behaviors
- Focus on at least one lifestyle component (physical activity, smoking, alcohol consumption, diet, stress, or sleep)
- Targeting vulnerable pregnant women, where vulnerability is defined as characteristics that stratify health opportunities and outcomes based on the PROGRESS-Plus framework; authors also had to specifically mention the characteristic as contributing to disparities in health opportunities or outcomes
- Presenting information on the design, reach, use, or implementation of the intervention
- Interventions taking place in a high-income country
- Full text available in English

**Exclusion criteria**

- Interventions targeting medically vulnerable women (eg, women with diabetes gravidarum or preeclampsia)
- Interventions not primarily administered during pregnancy
- Interventions consisting of <50% of eHealth components
- Interventions that were online advertisements or campaigns
- Case reports and reviews

### Data Extraction and Synthesis

We defined 4 phases based on 2 frameworks used in the process of eHealth intervention development, which we used as guides for data extraction and analysis. These were the design and use phases, derived from the Centre for eHealth Research Roadmap [31], and the reach and implementation phases, derived from the Reach, Effectiveness, Adoption, Implementation, and Maintenance framework [32]. For each article, AJPS extracted all strategies used and barriers encountered in the design, reach, use, or implementation phases, which was informed by the aforementioned frameworks. These strategies and barriers were then organized and presented into key themes for each phase. A strategy was classified as a facilitator if it was explicitly mentioned by the authors as beneficial to one or more phases of the intervention. If the authors suggested the strategy was possibly beneficial, it was referred to as a possible facilitator. Similarly, a factor was classified as a barrier if the authors explicitly mentioned it as detrimental to one or more phases of the intervention. When the authors suggested the factor as possibly detrimental, it was referred to as a possible barrier. If uncertainties arose regarding the phase that the strategy or encountered barrier belonged to or regarding its classification as a (possible) facilitator or barrier, discussions were conducted among the authors to address these uncertainties. In addition, we extracted information on the intervention components and theoretical frameworks used in the interventions. Furthermore, the study characteristics from the selected articles were organized in a Microsoft Excel file (Multimedia Appendix 3 [15,18-20,33-57]). We used a narrative synthesis to address our research question.

## Results

### Study Selection

**Figure 1:**
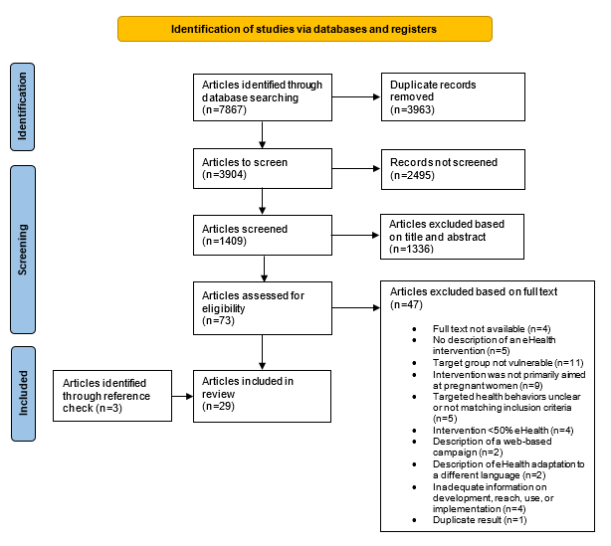
Flowchart of the article screening process.

The systematic search across the databases revealed 3904 potentially relevant citations. After screening 1409 titles and abstracts using ASReview, 73 (5.18%) articles were retained for full-text screening. A total of 36% (26/73) of these articles met the inclusion criteria and were included in this review, along with 3 articles that were identified through reference checking, resulting in 29 included articles (Figure 1).

### Description of the Included Studies

A summary of the characteristics of the 29 included articles is presented in Table 1. These 29 articles described 20 different interventions and included design papers (n=6, 21%), (pilot) randomized controlled trials (n=7, 24%), quasi-experimental studies (n=3, 10%), observational studies (n=4, 14%), protocols (n=2, 7%), a pilot evaluation (n=1, 3%), or a combination of study designs (n=6, 21%). These 20 interventions were delivered via apps (n=8, 40%), SMS text message (n=4, 20%), websites or web applications (n=3, 15%), tablets or computers (n=1, 5%), or a combination of modalities (n=3, 15%). For 5% (1/20) of the interventions, the modality had not been determined yet, but it was described as an app or digital tool [33]. A few eHealth lifestyle interventions (4/20, 20%) were combined with face-to-face or telephone coaching [34-38].

**Table 1:** Study characteristics (N=29).

Intervention name	eHealth modality used	Targeted health topic	Vulnerability factor and target group	Study and study design
Baby Steps to Healthier Habits or Baby Buddy	App	Diet and physical activity	SEP^a^—from economically and socially disadvantaged communities	Rhodes et al [45], 2023 (design paper)
Health-e Babies	App	Diet, physical activity, and stress	SEP—from socially disadvantaged communities	Dalton et al [19], 2018 (observational study)
Healthy Babies	SMS text messaging, web based, application and social media	Diet, physical activity, stress, and sleep	Ethnicity^b^—African American participants	Herring et al [37], 2019 (design paper and RCT^c^)
Healthy Pregnancy: Step by Step	Tablet or computer based program	Diet, stress, and smoking	SEP and ethnicity—underserved pregnant women	Mauriello et al [46], 2011 (quasi-experimental study) Prochaska et al [47], 2011 (design paper) Mauriello et al [15], 2016 (RCT)
I-PREGNO	App	Diet, physical activity, and stress	SEP, educational level, social capital, and age—low SEP or psychosocially burdened^d^	Vogel et al [38], 2023 (RCT protocol)
MAMA-EMPOWER	App	Diet, physical activity, stress, smoking, and alcohol consumption	Ethnicity—Aboriginal and Torres Strait Islander participants	Kennedy et al [48], 2021 (design paper, observational study and quasi-experimental study)
momHealth	Tablet and SMS text messaging	Diet, physical activity, and stress	Age—adolescents	Wambach et al [34], 2021 (quasi-experimental study) Wambach et al [35], 2022 (pilot RCT)
Mums and Bubs Deadly Diets	App or digital tool	Diet	Ethnicity—Aboriginal and Torres Strait Islander participants	Gilbert et al [33], 2023 (design paper)
My Healthy Pregnancy	App	Diet, stress, smoking, and alcohol consumption	SEP, ethnicity, and social capital—from a hard-to-reach population	Krishnamurti et al [49], 2017 (design paper, observational study and quasi-experimental study)
Quit4baby	SMS text messaging	Smoking	SEP—low income	Abroms et al [50], 2015 (observational study and quasi-experimental study) Abroms et al [42], 2017 (RCT) Leavitt et al [51], 2017 (observational study)
S.M.A.S.H. Out Cigarettes	Web based	Smoking	Age—adolescents	Comer and Grassley [52], 2010 (design paper)
SmartMoms in WIC^e^ or Healthy Beginnings	App	Diet, physical activity, stress, and sleep	SEP—low income and receiving WIC benefits	Flanagan et al [36], 2020 (design paper and RCT protocol)
SmokeBeat	App with wearable	Smoking	SEP—Medicaid eligible	Joyce et al [18], 2021 (pilot RCT)
Text4baby	SMS text messaging	Diet, physical activity, smoking, and alcohol consumption	SEP—low income	Whittaker et al [53], 2012 (design paper) Evans et al [39], 2012 (pilot evaluation) Remick and Kendrick [54], 2013 (design paper) Huberty et al [55], 2016 (design paper and observational study) Huberty et al [43], 2017 (RCT)
Together with Eva	App	Stress and smoking	SEP—low SEP	Van Dijk et al [56], 2021 (RCT protocol)
N/A^f^	Web based	Diet and physical activity	SEP—socially disadvantaged area	Carolan-Olah et al [57], 2021 (design paper and observational study)
N/A	Web based	Smoking	Place of residence and SEP—rural, economically depressed region	Harris and Reynolds [41], 2015 (pilot RCT)
N/A	SMS text messaging	Diet and physical activity	SEP—low income	Holmes et al [44], 2020 (RCT)
N/A	SMS text messaging (2 way)	Stress	SEP and ethnicity—low income and minority population	Song et al [20], 2013 (quasi-experimental study)
N/A	Social media and SMS text messaging	Diet, physical activity, and stress	SEP and age—adolescents and adults with a low income	Vander Wyst et al [40], 2019 (quasi-experimental study)

^a^SEP: socioeconomic position.

^b^Ethnicity, race, culture, and language.

^c^RCT: randomized controlled trial.

^d^Meeting at least one of the psychosocial burden factors as described in the study by Vogel et al [38].

^e^WIC: women and infant center.

^f^N/A: not applicable or unknown.

Most interventions (14/20, 70%) targeted multiple health behaviors. The most commonly targeted health behavior was diet (14/20, 70%), followed by physical activity (11/20, 55%), stress (11/20, 55%), smoking (9/20, 45%), alcohol consumption (3/20, 15%), and sleep (2/20, 10%). The included interventions addressed various vulnerabilities in pregnant women, and some (6/20, 30%) targeted multiple vulnerabilities. The most commonly targeted vulnerability was socioeconomic status (eg, low income; 15/20, 75%), followed by ethnicity (eg, Aboriginal and Torres Strait Islander participants; 6/20, 30%), age (adolescents; 3/20, 15%), social capital (eg, no partner; 2/20, 10%), place of residence (rural; 1/20, 5%), and educational level (low educational level; 1/20, 5%). The researchers of these interventions did not use low health literacy or limited digital skills as criteria for identifying the target group as vulnerable. Instead, low health literacy occasionally seemed to be considered as a mediator between vulnerability and the adoption of a healthy lifestyle and, therefore, necessitates attention in intervention design. Only 3% (1/29) of the studies reported the health literacy levels of their recruited participants [37].

Of the 29 studies, 10 (34%) investigated the efficacy or effectiveness of the intervention (Multimedia Appendix 3 [15,18-20,33-57]). These studies improved pregnancy-related knowledge [20], motherhood preparedness [39], eating behaviors [15], physical activity [35], and stress [20]. Some studies (3/29, 10%) showed mixed results in improving eating behaviors [40] and smoking cessation [41,42]. In addition, some interventions (5/29, 17%) failed to significantly improve their target group’s perceived uncertainty level [20], eating behaviors [35], physical activity [43], gestational weight gain [44], and smoking cessation [18]. To enhance effectiveness, several recommendations were proposed, including initiating the intervention earlier in pregnancy [44], increasing intervention duration [44], and integrating SMS text messaging as part of a multilevel intervention rather than relying exclusively on it [43].

### Design and Use

#### Overview

Table 2 highlights the strategies and barriers identified in the different phases of the eHealth lifestyle interventions. These phases involve design decisions, developing intervention content, and the willingness of individuals to use the intervention. In this section, we elaborate on these strategies to provide a detailed overview of the findings.

**Table 2:** Strategies and barriers per phase. Symbols are used to indicate whether authors mentioned a strategy as a facilitator (++), possible facilitator (+), possible barrier (–), barrier (– –), or facilitator and possible barrier (++ -). The absence of a symbol means that the strategy was used, but that the authors did not mention the strategy to be a (possible) facilitator or (possible) barrier.

Phase and theme		Example	Studies
**Design and use**			
	Adaptation of or extension to an existing (eHealth) intervention	Adaptations were made to the previous intervention to better meet the needs of economically disadvantaged women [36].	Flanagan et al [36] Vogel et al [38]++ Abroms et al [42]++ Rhodes et al [45]++ Mauriello et al [46] Abroms et al [50]+ Huberty et al [55]+ Leavitt et al [51]
	Collaborating with stakeholders	Research began by consulting with 4 medical expert informants in the field of maternal-fetal medicine and community informants from a diverse set of groups (eg, churches, nonprofit organizations, women’s shelters, and doula groups) [49].	Mauriello et al [15]++ Joyce et al [18]++ Dalton et al [19]++ Song et al [20]++ Gilbert et al [33]++ Wambach et al [34]++ Flanagan et al [36]++ – Herring et al [37]++ Vogel et al [38]++ Abroms et al [42]++ Rhodes et al [45]++ Mauriello et al [46]++ Prochaska et al [47]++ Kennedy et al [48]++ Krishnamurti et al [49]++ Abroms et al [50]++ Whittaker et al [53]++ – Remick and Kendrick [54]++ Huberty et al [55]++ Van Dijk et al [56]++ Carolan-Olah et al [57]++
	Financial aspects	Limitations on app development and research were budgetary constraints due to the level of pilot funding [48].	Mauriello et al [46]– – Kennedy et al [48]– –
	Preventing attrition	To address attrition after enrollment, the recruitment staff was trained to focus on clear, unrushed explanations of the study requirements during the invitation and consent processes [34].	Mauriello et al [15]++ Wambach et al [34]+
	Providing devices	To assist with adherence, participants were provided with a digital “bathroom” scale for self-weighing [37].	Joyce et al [18] Song et al [20] Wambach et al [34]– – Wambach et al [35] Flanagan et al [36] Herring et al [37]+ Harris and Reynolds [41] Huberty et al [43]
	Technical problems	Technology issues included limited internet connectivity during teleconference meetings [34].	Joyce et al [18]– – Dalton et al [19]– – Wambach et al [34]– – Kennedy et al [48]– –
	Offering technological support	The study coordinator checked how the smartwatch and app were functioning and helped with any technical issues encountered [18].	Joyce et al [18]+ Dalton et al [19]+ Wambach et al [34,35]+
	Tailoring	If the app detected a decrease in self-reported cigarette use, it provided encouraging messages in addition to quitting resources [49].	Mauriello et al [15]+ Joyce et al [18] Song et al [20]+ Gilbert et al [33] Flanagan et al [36]+ Herring et al [37]+ Vander Wyst et al [40] Holmes et al [44]+ Mauriello et al [46]+ Prochaska et al [47] Kennedy et al [48]+ Krishnamurti et al [49] Comer and Grassley [52]+ Whittaker et al [53]+ Remick and Kendrick [54]+ Van Dijk et al [56]+ Carolan-Olah et al [57]+ –
	Using incentives	Incentive payments were earned for consistent smartband wearing and abstaining from smoking [18].	Mauriello et al [15] Joyce et al [18] Flanagan et al [36] Harris and Reynolds [41]+ Krishnamurti et al [49]
	Using theoretical frameworks	The website was developed using elements of social cognitive theory [57].	Mauriello et al [15]++ Gilbert et al [33]++ Wambach et al [34,35]++ Herring et al [37]++ Evans et al [39]++ Vander Wyst et al [40]++ Harris and Reynolds [41] Abroms et al [42]++ Huberty et al [43] Holmes et al [44]++ Rhodes et al [45]++ Mauriello et al [46]++ Prochaska et al [47]++ Kennedy et al [48] Krishnamurti et al [49]++ Abroms et al [50]++ Comer and Grassley [52]++ Carolan-Olah et al [57]++
	Using clinical and best practice guidelines	One of the modules was based on the most recent best practice guidelines on physical activity and exercise during pregnancy [57].	Harris and Reynolds [41]++ Abroms et al [42,50]++ Comer and Grassley [52]++ Carolan-Olah et al [57]++
**Reach**			
	Access to the internet and devices	Women with non-Android mobile phones were excluded due to the app having been developed for Android smartphones only [19].	Dalton et al [19]– – Evans et al [39]– – Harris and Reynolds [41] Prochaska et al [47]
	Collaborating with stakeholders	Limited clinical staff support was experienced for assisting research personnel during recruitment visits [34].	Mauriello et al [15]++ Joyce et al [18]++ Song et al [20]++ Wambach et al [34]– – Herring et al [37]++ Vogel et al [38]+ Evans et al [39]– – Harris and Reynolds [41]++ Mauriello et al [46]++ Prochaska et al [47]++ Kennedy et al [48]++ Whittaker et al [53]++ Van Dijk et al [56]++
	Reluctance to participate	Some potential participants had misgivings about enrolling in a service that involved providing their mobile phone number and other personal information, such as their baby’s due date [39].	Joyce et al [18]– – Wambach et al [34]– – Evans et al [39]– –
	Limited number of eligible participants	Due to the high rate of preterm labor in this population, recruiting women in their second and third trimesters proved to be challenging [20].	Mauriello et al [15]– – Joyce et al [18]– – Song et al [20]– – Wambach et al [34]– –
	Using incentives	Removal of the reference to the incentive in the recruitment message significantly reduced response and enrollment [51].	Joyce et al [18] Song et al [20] Gilbert et al [33] Flanagan et al [36] Herring et al [37] Vogel et al [38] Abroms et al [42] Huberty et al [43] Rhodes et al [45] Mauriello et al [46] Krishnamurti et al [49] Abroms et al [50] Leavitt et al [51]++ Huberty et al [55] Van Dijk et al [56] Carolan-Olah et al [57]
**Implementation**			
	Collaborating with stakeholders	The involvement of diverse national public, private, and local partners was vitally important for national uptake [53].	Mauriello et al [15]+ Flanagan et al [36]+ Vogel et al [38]+ Prochaska et al [47]+ Whittaker et al [53]++
	Financial aspects	The funding from and involvement of high-profile national partners made an aggressive timeline to national launch possible [53].	Harris and Reynolds [41]– Mauriello et al [46]++ Whittaker et al [53]++ Remick and Kendrick [54]++
	Integration into health care	Once developed, the intervention was easy to implement in the health care system [56].	Flanagan et al [36] Vogel et al [38] Mauriello et al [46] Prochaska et al [47] Krishnamurti et al [49] Comer and Grassley [52] Van Dijk et al [56]
	Low workload	The intervention did not require much from WIC^a^ staff as this is commonly acknowledged as a roadblock for the implementation of long-standing programs [36].	Flanagan et al [36]++ Harris and Reynolds [41]++ Mauriello et al [46]++
	Mobile optimization	Mobile optimization allowed the program to be distributed via any internet-enabled device.	Mauriello et al [15]++

^a^WIC: women and infant center.

#### Adaptation of or Extension to an Existing (eHealth) Intervention

Some of the eHealth interventions included components of existing interventions or were adaptations of existing interventions. For instance, SmartMoms in WIC [36], Quit4baby [42], I-PREGNO [38], and Healthy Pregnancy: Step by Step [46] made adaptations to existing interventions to better meet the specific needs of their vulnerable target groups. They made these adaptations, such as creating a sense of community, by including support groups through Facebook based on recommendations provided by stakeholders [36,46]. In addition, Rhodes et al [45] and Huberty et al [55] developed their interventions as an extension to existing eHealth interventions or modalities and referred to existing eHealth modalities as well suited for the implementation and evaluation of novel eHealth lifestyle interventions.

#### Theoretical Frameworks and Guidelines

Over half (17/29, 59%) of the studies used theoretical frameworks for behavior change, which contributed substantially to the design of their interventions. The frameworks used were social cognitive theory [37,39,40,42-44,50,52,57], the transtheoretical model of behavior change [15,39,41,46,47], the multiple health behavior change paradigm [34,35], the Behavior Change Wheel [48], the Fogg behavior model [45], and the health belief model [39]. Most studies (24/29, 83%) did not provide justifications for their selection of a particular behavior change framework. However, Kennedy et al [48] and Mauriello et al [15] selected their frameworks based on their effectiveness in previous studies. In addition, the Behavior Change Wheel was used for its ability to advance understanding of features in need of improvement [48]. The multiple health behavior change paradigm was chosen as a guiding framework due to its unique approach in addressing multiple health behaviors simultaneously through one intervention [34,35]. In addition, the researchers used clinical and best practice guidelines to inform the content of their interventions [41,42,50,52,57]. Furthermore, theoretical frameworks and guidelines were used to guide the development of the eHealth tools, including the Centre for eHealth Research Roadmap [48], the behavioral decision research paradigm [49], the Behavior Change Wheel [45], the Sanders and Stappers co-design framework [33], the Kaupapa Māori framework [33], and Noorbergen’s guidelines for co-design of mobile health (mHealth) systems [33].

#### Collaborating With Stakeholders

Stakeholders provided valuable guidance to researchers in terms of the design and content of the eHealth lifestyle interventions. Stakeholders included, among others, the target group, research centers, academics specialized in different health domains, social service providers, literacy experts, and mHealth companies. For co-creation with the target group, the researchers used multiple methods, such as surveys [20,55], interviews [18,37,45,47-49,55,57], focus groups [19,37,38,45-47,53,56], user research [45-49], and monthly meetings [36], to ensure that their target group’s needs were met in terms of design, literacy, content, and usability of the eHealth lifestyle intervention. For instance, findings from interviews with the target group can indicate barriers that women encounter related to healthy lifestyle practices during pregnancy [57]. In turn, this knowledge can inform the design of an eHealth lifestyle intervention that overcomes these barriers.

However, collaborating with stakeholders can also entail challenges. For example, Flanagan et al [36] experienced a disparity between their mothers advisory group’s request to include more health markers in their trial and the limited enthusiasm for this adaptation from the scientific review panel. Similarly, Whittaker et al [53] encountered challenges in information sharing and estimating the in-kind costs of their initiative. They also faced confusion regarding defined roles and responsibilities among their partners and differing perspectives and priorities in transitioning to the next stage of their partnerships. Establishing and adhering to a set of guiding principles, comprising key intervention design objectives and features, could ensure that all stakeholders involved in the intervention design work toward a common vision [45].

#### Tailoring

##### Overview

Many studies (17/29, 59%) tailored their eHealth lifestyle intervention to the characteristics and skills of their target group, for example, by matching the content of their intervention to their target groups’ norms and values or literacy level. Some interventions (9/29, 31%) provided individualized tailored information or feedback based on gestation [53], collected participant data (eg, body weight measurements) [18,36,46,49], stage of change [15,37,46], or the Behavior Change Wheel [48]. In some studies (2/29, 7%), the researchers mentioned tailoring of tips, recipes, and feedback without providing details about what the tailoring was based on [37,47].

##### Language and Culture

Certain researchers made sure that their intervention was available or would become available in multiple languages [15,46,53,54], whereas others paid special attention to the level of (health) literacy of their target group [18,20,36,40,44,46,52,54,56,57]. The importance of health literacy and language availability in eHealth interventions was highlighted by several studies. For instance, Carolan-Olah et al [57] used multiple photos and illustrations, limited textual content, and maintained a single idea per slide in their intervention to improve access for women with low levels of health literacy. However, some of the less educated women found that there was still too much information provided in the modules. In addition, their intervention was solely available in English, and the women expressed a preference for it to be available in other languages as well. In addition, in their intervention, Evans et al [39] found that educational level was an important factor for health belief outcomes and suggested that this could be a result of differences in literacy levels and message comprehension.

Culture was incorporated into the content and design of the interventions to increase its relevance to or acceptability by the target group [15,36,46,52], align their messages with the norms and values of the target group [33,36,54], and cater to the preferences expressed by the target group [48]. However, most of these studies (4/7, 57%) did not describe in detail how culture was incorporated into their intervention. Nevertheless, in some interventions (3/7, 43%), researchers included images of women in the same age categories and from the same ethnic backgrounds as their target group [46,48,52]. Furthermore, recipes and links were provided from specific cuisines to align with the high proportion of particular ethnic groups in the area in which recruitment took place [57].

#### Providing Devices

Researchers provided devices to their participants for various purposes. Some researchers provided devices necessary for the delivery of the intervention [18,34,35], whereas others provided devices to collect data for evaluating the intervention’s effectiveness. The latter included a Fitbit to measure physical activity [36,43], a pedometer to track step counts [37], a piCO Smokerlyzer to measure breath carbon monoxide [41], and a digital scale to monitor gestational weight gain [27,37]. In addition to evaluating effectiveness, Herring et al [37] highlighted that providing devices might facilitate adherence to the intervention. However, personal reasons can influence the use of devices, such as not being able to wear a smartwatch during work [18].

#### Technical Problems and Offering Support

Technical problems were expressed by participants in several studies (4/29, 14%) [18,19,34,48], often negatively influencing engagement. Multiple studies (4/29, 14%) provided technological support to prevent or help with technological issues [18,19,34,35]. For example, information was provided on how to use the device, download the app, and synchronize devices, and contact information for study coordinators was provided to help when the participant encountered technical issues. However, offering contact details for technological support alone may not be enough. For example, Dalton et al [19] provided phone numbers for technological support but found that 9% of the participants failed to report the problems they encountered while downloading the app. Leaving participants to manage the app on their own to assess its usability might have influenced the high dropout rate in their study [19].

#### Preventing Attrition

In a few studies (4/29, 14%), the researchers mentioned attrition of participants after enrollment [18,19,34,35]. Reasons that might have contributed to this attrition were participants’ employment status [19], financial constraints [19], anxiety levels [19], and time constraints [18,19,34]. To retain participants, Mauriello et al [15] had participants engage with the intervention during their appointments, contacted participants who could not be reached in various ways to make them complete final assessments, and used incentives for completing a session. These strategies resulted in an impressive retention. The attrition in the study by Wambach et al [34] prompted the development of strategies to limit attrition in future research. These included enhancing staff training to focus on a clear and unrushed explanation of study requirements during recruitment, improving the description of study requirements on an advertising flyer, and including larger incentives. This resulted in a lower attrition rate, although it remained high at 25.8% [35].

#### Features Used in eHealth Interventions

The features (components that make up the eHealth intervention) included in the eHealth interventions are summarized in Table 3. Many interventions (6/20, 30%) included a feature in which participants could interact with others in their intervention, such as a chat room or Facebook page [19,34-37,48,52]. However, in one intervention, peer support was considered the least useful component by most of the participants [34]. Some interventions (2/20, 10%) actively included partners as a way of providing social support to pregnant women [38,45]. In addition, links to external sources of information were provided, which prevented the inclusion of too much information in the content of the intervention but also catered to those who were looking for more information about a certain topic [19,34,35,40,41,43,45,52,55,57]. Furthermore, although some interventions included weight trackers, Rhodes et al [45] decided not to include weight monitoring or weight-related messages based on their potential to demotivate their participants.

Some of the incorporated features used were specific to a limited number of interventions. For example, Krishnamurti et al [49] included Uber services in their intervention to provide free transportation to prenatal care appointments as transportation was revealed to be a barrier for their target group. Providing Uber transportation prevented missed appointments and was found to be cost saving. Song et al [20] were the only ones to include an automated, 2-way SMS text messaging system in their intervention to distribute pregnancy and health-related information and foster patient–health care provider interaction. Despite some frustrations regarding its ability to answer participants’ questions, the SMS text messaging system could promote health communication while offering psychological benefits as well [20]. However, it was suggested that the addition of more system-initiated SMS text messages could benefit women who are less comfortable with asking questions [20].

**Table 3:** Features of the eHealth lifestyle interventions.

Features	Studies
Calculator	Mauriello et al [15] Kennedy et al [48] Carolan-Olah et al [57]
Diary	Vogel et al [38] Van Dijk et al [56]
Feedback	Mauriello et al [15] Flanagan et al [36] Joyce et al [18] Rhodes et al [45] Mauriello et al [46] Kennedy et al [48] Krishnamurti et al [49] Comer and Grassley [52]
Food serving size measurements	Carolan-Olah et al [57]
Fictitious peer offering advice	Abroms et al [42]
Game or quiz component	Wambach et al [35] Flanagan et al [36] Herring et al [37] Abroms et al [42] Carolan-Olah et al [57]
Goal setting	Mauriello et al [15] Herring et al [37] Vogel et al [38] Abroms et al [42] Rhodes et al [45] Kennedy et al [48] Carolan-Olah et al [57]
Motivation from peers	Abroms et al [42]
Multimedia	Wambach et al [34,35] Flanagan et al [36] Vogel et al [38] Vander Wyst et al [40] Rhodes et al [45] Mauriello et al [46] Prochaska et al [47] Kennedy et al [48] Comer and Grassley [52] Carolan-Olah et al [57]
Links to external sources of information	Dalton et al [19] Wambach et al [34,35] Vander Wyst et al [40] Harris and Reynolds [41] Huberty et al [43] Rhodes et al [45] Comer and Grassley [52] Huberty et al [55] Carolan-Olah et al [57]
Messages of support	Mauriello et al [15]
Pregnancy-tracking features	Dalton et al [19] Kennedy et al [48] Krishnamurti et al [49]
Real-time alerts to medical staff	Krishnamurti et al [49]
Recipes	Mauriello et al [15] Flanagan et al [36] Herring et al [37] Vander Wyst et al [40] Harris and Reynolds [41] Rhodes et al [45] Kennedy et al [48] Carolan-Olah et al [57]
Reminders	Dalton et al [19] Abroms et al [42] Rhodes et al [45] Krishnamurti et al [49] Remick and Kendrick [54]
Self-assessment and self-monitoring	Flanagan et al [36] Herring et al [37] Vogel et al [38] Rhodes et al [45] Kennedy et al [48] Krishnamurti et al [49]
Social component	Wambach et al [34,35] Flanagan et al [36] Herring et al [37] Vogel et al [38] Rhodes et al [45] Dalton et al [19] Kennedy et al [48] Comer and Grassley [52]
Stress-reducing exercises	Dalton et al [19] Vogel et al [38] Mauriello et al [46] Kennedy et al [48] Van Dijk et al [56]
Free transportation	Krishnamurti et al [49]
Two-way SMS text messaging system	Song et al [20]
Weight tracker	Flanagan et al [36]

### Reach

Table 2 highlights the strategies and encountered barriers identified in the reach phase, which involves recruitment methods and the willingness of individuals to participate in the intervention. In this section, we elaborate on the strategies found and barriers encountered.

#### Recruitment Characteristics

In the included studies, the researchers used multiple sites and strategies and involved various key persons to recruit vulnerable pregnant women (Table 4). Often, recruitment took place in a health care setting (19/29, 66%), and many studies (12/29, 41%) received support from health professionals for recruitment. Apart from face-to-face or phone recruitment (22/29, 76%), many studies used printed materials (11/29, 38%) or the internet (5/29, 17%) to promote their interventions. Printed materials were placed at sites frequently visited by pregnant women, such as schools and children’s retail stores.

**Table 4:** Recruitment characteristics.

Recruitment characteristic			Studies
**Individuals involved**			
	Health care professionals	Mauriello et al [15] Herring et al [37] Vogel et al [38] Song et al [20] Evans et al [39] Harris and Reynolds [41] Mauriello et al [46] Prochaska et al [47] Comer and Grassley [52] Whittaker et al [53] Van Dijk et al [56]	
	Researchers	Gilbert et al [33] Wambach et al [34] Flanagan et al [36] Herring et al [37] Holmes et al [44] Kennedy et al [48] Whittaker et al [53] Huberty et al [55] Carolan-Olah et al [57]	
	Staff from non–health care organizations	Joyce et al [18] Kennedy et al [48] Whittaker et al [53]	
	Professional recruitment services	Rhodes et al [45]	
**Recruitment sites**			
	Health care setting	Mauriello et al [15] Joyce et al [18] Dalton et al [19] Gilbert et al [33] Wambach et al [34,35] Herring et al [37] Vogel et al [38] Evans et al [39] Vander Wyst et al [40] Harris and Reynolds [41] Huberty et al [43] Mauriello et al [46] Prochaska et al [47] Kennedy et al [48] Krishnamurti et al [49] Huberty et al [55] Van Dijk et al [56] Carolan-Olah et al [57]	
	Federal benefit and assistance clinics for low-income women and families	Flanagan et al [36] Herring et al [37] Holmes et al [44]	
	Public places (schools, family support centers, churches, retail stores, and events)	Wambach et al [35] Huberty et al [43] Krishnamurti et al [49]	
	Homes of the target group	Song et al [20]	
	Community organizations	Gilbert et al [33] Kennedy et al [48]	
**Recruitment strategies**			
	Personal contact or phone call	Mauriello et al [15] Joyce et al [18] Dalton et al [19] Gilbert et al [33] Wambach et al [34,35] Flanagan et al [36] Herring et al [37] Vogel et al [38] Evans et al [39] Vander Wyst et al [40] Harris and Reynolds [41] Holmes et al [44] Mauriello et al [46] Prochaska et al [47] Krishnamurti et al [49] Whittaker et al [53] Huberty et al [55] Van Dijk et al [56] Carolan-Olah et al [57]	
	Online (social media, websites, or discussion boards)	Huberty et al [43] Rhodes et al [45] Whittaker et al [53] Huberty et al [55] Van Dijk et al [56]	
	Printed materials (flyers, posters, and brochures)	Gilbert et al [33] Wambach et al [34,35] Flanagan et al [36] Vander Wyst et al [40] Harris and Reynolds [41] Huberty et al [43] Kennedy et al [48] Whittaker et al [53] Huberty et al [55] Van Dijk et al [56]	
	Email (listserve) or SMS text messaging	Huberty et al [43,55]	
	Word-of-mouth or grassroots strategies	Wambach et al [34] Harris and Reynolds [41] Huberty et al [43] Kennedy et al [48] Huberty et al [55]	
	Existing eHealth interventions	Abroms et al [42] Rhodes et al [45] Leavitt et al [51]	
	A clinical study or pregnancy support program	Joyce et al [18] Vander Wyst et al [40]	
	Home visits	Song et al [20]	
	Personal network	Kennedy et al [48]	
	Survey	Rhodes et al [45]	

#### Recruitment Challenges and Strategies

Challenges related to participant recruitment were frequently encountered. One of these challenges was a limited number of eligible participants [15,18,20,34]. For example, Song et al [20] experienced challenges in recruiting pregnant women in their second and third trimesters due to a high rate of preterm labor within the low-income minority pregnant population. A second challenge in recruitment was due to eligible candidates’ reluctance to participate because of a lack of interest, time constraints, and concerns about sharing personal information [18,34,39]. Health care professionals and staff from non–health care organizations were often mentioned as facilitators in recruiting the target group, either providing assistance or taking full responsibility for the recruitment process [15,18,20,37,41,46-48,53,56], but sometimes this posed challenges as well [34,39]. For example, research personnel in the study by Wambach et al [34] received limited support from clinical staff during recruitment visits. Finally, resource aspects, including limited phone ownership [39], lack of access to an internet service [19,47], and phone operating systems that were incompatible with the eHealth intervention [19], hindered recruitment. In some studies (6/29, 21%), recruitment difficulties resulted in a small sample size [20,34,39-41,48].

Certain strategies were implemented to overcome recruitment challenges. For example, in response to the limited number of pregnant adolescents, Wambach et al [34] expanded their age range and the number of recruitment sites and included word-of-mouth recruitment. Second, in the study by Harris and Reynolds [41], research personnel conducted home visits to set up equipment and provide detailed training on its use, thereby enhancing accessibility for rural pregnant smokers. Furthermore, to overcome participants’ limited access to an internet or telephone service, Prochaska et al [47] encouraged providers of the intervention to have a computer kiosk at their centers for women to access the program.

#### Incentives

Many studies (18/29, 62%) offered incentives to encourage participation in their trial, interview, focus group, or workshop [15,20,33,43,45,46,51,55,57]; attend study visits or video check-ins [18,36]; complete assessments, questionnaires, or interviews [20,38,49,50,56]; submit self-monitoring data [37] or bodily samples [42]; engage with the intervention [15,18,36,49]; or abstain from unhealthy behaviors [18,41]. The incentives provided in these studies included gift cards [15,20,33,36,37,41,42,46,50,57]; monetary compensation [38,41,45,49,55]; devices such as smartphones [20,49], scales [49], and smartwatches [43]; and health-related items such as yoga mats and prenatal vitamins [36].

Few studies (2/18, 11%) discussed the impact of the incentives they used. Nevertheless, Leavitt et al [51] observed a significant decline in the response rate after removing the reference to their incentive in their recruitment message. Furthermore, Harris and Reynolds [41] believed that their incentives enhanced participants’ motivation to quit smoking by the specified quit date. However, in the study by Joyce et al [18], qualitative interviews revealed mixed feelings toward financial incentives, where one participant stated that the financial incentive was encouraging to quit smoking, whereas another participant expressed that a motivational tailored message would be rewarding enough without financial rewards.

### Implementation

#### Overview

Assessment of implementation includes factors such as the successfulness and costs of intervention delivery. Most studies (18/29, 62%) did not describe how their eHealth lifestyle intervention was or would be implemented. However, some strategies in the implementation phase of the eHealth lifestyle interventions were identified, which are highlighted in Table 2.

#### Financial Aspects

A limited number of studies (5/29, 17%) described whether costs and funding acted as barriers or facilitators in the design or implementation phase of their eHealth lifestyle intervention. Nevertheless, in 7% (2/29) of the studies, budget constraints limited intervention development [46,48]. Furthermore, costs from financial incentives and loaned devices could be barriers to intervention implementation [41]. Implementing the intervention in a clinical setting was seen as a way to reduce costs [41]. Whittaker et al [53] mentioned how funding from and involvement of well-known national partners facilitated the national launch of their intervention. In addition, providing the intervention free of charge to vulnerable pregnant women could facilitate implementation [53,54].

#### Dissemination and Integration Into Health Care Structures

A few strategies were highlighted in the studies to ensure the dissemination of their interventions. First, collaboration with stakeholders was frequently emphasized as vital for the successful implementation of the interventions. For example, the adoption of an intervention by a governmental program or by existing perinatal care services can facilitate widespread implementation and distribution [36,38]. The enthusiasm of stakeholders and the intervention’s relevance to them were mentioned as characteristics that should contribute to the ease of the dissemination of their interventions [15,47]. Second, a low workload for intervention deliverers was mentioned in multiple studies (3/29, 10%) as an important aspect of a feasible implementation [36,41,46]. Third, providing the intervention in a format that can be distributed via any internet-enabled device, either in health care settings or via a personal internet-enabled device at the convenience of its user, contributes to dissemination [15]. Furthermore, media appearance can result in a considerable increase in rates of enrollment in the intervention [53]. Finally, to develop an intervention that could be easily integrated into health care structures, dissemination issues should be considered from the start of the project [47].

A few research groups developed their interventions with the aim of integrating them into the current health care structures [36,38,46,47,49,52,56]. Several suggestions were given for this integration, such as women using the intervention before their consult and sharing a printed report of their results with their health care provider [47]. For this integration, it was important that the program be self-directed, require little to no staff training, and allow for low-cost and consistent delivery [46]. However, taking health care providers’ time and resource constraints into consideration, an intervention can be intentionally developed to be used outside of health care as well [37].

## Discussion

### Principal Findings

#### Overview

This scoping review provides a comprehensive overview of the applied strategies in 4 phases (design, reach, use, and implementation) of eHealth lifestyle interventions for vulnerable pregnant women. In addition, it highlights which barriers researchers encountered and which strategies acted as facilitators for these interventions. By identifying barriers and facilitators in current eHealth lifestyle interventions aimed at vulnerable pregnant women, our study generated insights into how to optimize eHealth lifestyle interventions for this population. As this scoping review covered a wide range of interventions, study designs, targeted health behaviors, and vulnerabilities, it also contributes to a nuanced understanding of the landscape of available eHealth lifestyle interventions for vulnerable pregnant women.

We included 29 articles describing 20 eHealth interventions in this review. These interventions were delivered through different modalities, targeted different lifestyle components, and were aimed at pregnant women with different vulnerabilities. The studies examining the effectiveness of the eHealth lifestyle interventions showed potential as some managed to significantly change health behaviors, but the results were inconsistent. This finding aligns with those of previous research on eHealth lifestyle interventions for the low-SEP population [58], showing that effect sizes are small and differ among the interventions. This enhances the need for insights into the experienced barriers and facilitators in the different phases of the development of an eHealth intervention. The insights gained from our review will be explored separately per phase.

#### Intervention Design and Use

For the design of eHealth lifestyle interventions, researchers were guided by stakeholders, existing health interventions, guidelines, and theoretical frameworks. Researchers that collaborated with their target group and other stakeholders (eg, health care professionals) often referred to these collaborations as facilitating to their intervention design [15,18-20,33,34,36-38,42,45]. While co-creating with the target group was mostly limited to content design of the intervention, other stakeholders were often involved in multiple phases of intervention development. Engaging in qualitative research with intended users can shape the foundational guiding principles of the intervention, and user feedback can help refine the content and functionality of the intervention [45]. Existing health interventions were examined to identify components that could be valuable for the design of new eHealth interventions [36,38,42,45,46,50,55,56]. For clinical content, researchers additionally consulted guidelines from organizations such as the American Congress of Obstetricians and Gynecologists [41]. In addition, various theoretical frameworks for behavior change were used to guide the content of the interventions. However, it often remained unclear why researchers chose the frameworks they used. Furthermore, even though frameworks could help guide health intervention design, evaluation, adaptation, and implementation, only some studies (4/29, 59%) mentioned using a framework to guide the development of their eHealth tool.

Various features and strategies were included in the eHealth lifestyle interventions to increase user engagement. First, including social components (eg, virtual peer support sessions and partner involvement) in the intervention was (expected to be) appreciated by end users [19,34-38,45,48,52]. Research indicates that pregnant women value partner involvement and support in eHealth lifestyle interventions and its benefits could extend beyond improving health outcomes [59]. Nevertheless, this review revealed a limited number of interventions (2/20, 10%) that actively involved partners to support maternal health behavior. Future eHealth lifestyle interventions should explore ways of engaging partners of vulnerable pregnant women as their involvement seems promising. Furthermore, although it was somewhat unclear whether tailoring led to better results in the included studies, tailoring the intervention to the characteristics and needs of the target group or individuals is expected to lead to increased engagement [60]. The same expectation applies to the use of incentives [61]. Technological support was provided to assist end users with any technological difficulty encountered. Although positive assumptions about pregnant women’s digital literacy due to their age may be made, one study highlighted an instance in which technical challenges adversely affected user engagement [19]. In addition, digital literacy has previously been identified as a barrier to mHealth adoption among people of a low socioeconomic status [62]. These results highlight the importance of addressing digital literacy when developing eHealth interventions for vulnerable pregnant women.

#### Reaching Vulnerable Pregnant Women

Recruitment of vulnerable groups for study purposes frequently presents challenges, as has been emphasized in a previous review [58] and was once more highlighted in this review, where most researchers encountered difficulties in the recruitment of vulnerable pregnant women for participation in their studies to test their eHealth lifestyle interventions. In a few studies (6/29, 21%), difficulties with recruitment resulted in a small sample size [20,34,39-41,48]. However, the researchers did not always explicitly discuss the barriers that kept them from achieving an adequate sample size. Interestingly, our findings show that recruitment barriers were not solely attributed to factors such as low patient volumes or lack of interest by participants. Health care providers, although often identified as facilitators in the recruitment process, were also identified as barriers, either because the research staff received limited support from health care providers or because recruitment was not feasible for health care providers operating in their natural setting [34,39]. Finally, resource aspects (eg, lack of access to internet and limited phone ownership) were mentioned as a barrier to the recruitment of vulnerable pregnant women [19,39,47].

The study by Mauriello et al [46] was the only one that not only met but also exceeded its recruitment goals within a short time frame, and they attributed this success to the willingness of the prenatal care staff and eagerness of the pregnant women attending the health center where they recruited from. However, it remains unclear what exactly caused this eagerness. Many studies (11/29, 38%) involved health care professionals in the recruitment process, whereas community-led recruitment was less prevalent. However, using a community-based participatory research approach has shown to be a promising strategy for conducting health disparity–related research in minority populations [63] and, therefore, might be considered in the design of future interventions targeting vulnerable pregnant women. In addition, including incentives could facilitate recruitment, although we only found one study that clearly showed that their reference to an incentive increased recruitment numbers [42,51].

Overall, to increase the successful recruitment of vulnerable pregnant women, researchers should consider all 4 conditions that were identified as barriers to recruitment in this review. First, it is important to identify the prevalence of the target group, and recruitment sites should be adjusted accordingly based on this prevalence. Second, researchers ought to incorporate methods to ensure that participation in eHealth interventions is appealing and minimally time-consuming for their target group. Including incentives could be particularly helpful in addressing this concern. Third, researchers should aim to either handle recruitment themselves or involve dedicated health care providers in the recruitment process, ensuring it does not become resource intensive or time-consuming for them or disrupt the delivery of health care. Finally, resource-related barriers to recruitment can be handled by enabling access to internet services during recruitment and providing the devices necessary for the use of the eHealth lifestyle intervention.

#### Implementation

The insights from the facilitators and barriers encountered in previous eHealth implementation processes could help future researchers, health care professionals, and eHealth developers devise more effective strategies for forthcoming implementations. Nevertheless, most studies (18/29, 62%) failed to outline the implementation process of their eHealth lifestyle intervention. This could be attributed to the prevalence of pilot and design papers among the included studies, where the interventions had often not been implemented beyond these initial stages. However, a few facilitators could be identified related to implementation. First, collaboration with stakeholders was frequently emphasized as necessary for a successful implementation of the intervention [15,36,38,47,53]. Collaborating with stakeholders enhances the likelihood that interventions are designed and implemented in a manner that is relevant, acceptable, and feasible within real-world settings [64,65]. In addition, providing the intervention free of charge was important for uptake by end users. Furthermore, as eHealth lifestyle interventions often include the involvement of health care professionals, for whom time constraints can be a barrier to involvement, a low workload came forward as an important facilitator to eHealth implementation. In contrast, the costs associated with financial incentives and loaned devices were found to be a potential barrier to implementation.

### Limitations

This scoping review is the first to identify strategies in the development of eHealth lifestyle interventions for vulnerable pregnant women. The barriers and facilitators that were identified can guide researchers, health care professionals, and eHealth developers in the development of future eHealth tools for this target group. However, this review has some limitations. Although certain strategies emerged as clear facilitators, the authors did not consistently report on the impact of other applied strategies. This lack of reporting on facilitators and barriers within specific interventions was also noted in an earlier review about eHealth lifestyle interventions in the low-SEP population [58]. Identifying strategies and obstacles encountered as possible barriers and facilitators allowed us to partly solve this limitation. Guidelines that ensure complete and accurate documentation of eHealth development and implementation, such as the Guidelines and Checklist for the Reporting on Digital Health Implementation, foster the transparency necessary for future developers and, therefore, should be used when reporting on eHealth development and implementation [66]. In addition, facilitators were mentioned more often than barriers in the articles, which might be related to publication bias, in which articles about interventions that failed are not written or published. Furthermore, the heterogeneity of the lifestyle behaviors and eHealth modalities in the included studies might limit the generalizability of the barriers and facilitators to other lifestyle behaviors and eHealth modalities.

### Conclusions

This scoping review provides a comprehensive overview of the strategies used and the challenges faced in developing and implementing eHealth lifestyle interventions for vulnerable pregnant women throughout different phases of development. Specifically, our findings in the design and use phases of eHealth lifestyle interventions highlight the importance of stakeholder engagement, a user-centered design, theoretical frameworks, tailoring to the needs and skills of the target group, and providing technological support. Furthermore, the challenges and strategies related to recruitment underscore the complexities involved in engaging vulnerable populations in research and interventions. By identifying barriers such as limited access to resources and health care provider support, this scoping review offers practical recommendations for improving the reach of vulnerable pregnant women. The insights into implementation facilitators and barriers highlight the importance of stakeholder collaboration, a low workload for intervention deliverers, and financial considerations. These findings provide valuable guidance for researchers, health care professionals, and eHealth developers seeking to implement eHealth interventions effectively within health care settings and broader community contexts.

Despite the considerable insights derived from this review, more detailed reporting on the impact of the strategies used and barriers encountered in eHealth lifestyle interventions for vulnerable pregnant women is warranted. Nevertheless, our insights will pave the way for the development of more impactful eHealth interventions for vulnerable pregnant women, ultimately enhancing the health of both mothers and their offspring.

## Appendix

Multimedia Appendix 1 PRISMA-ScR (Preferred Reporting Items for Systematic Reviews and Meta-Analyses extension for Scoping Reviews) checklist.

Multimedia Appendix 2 Search strategy.

Multimedia Appendix 3 Study characteristics.

## Abbreviations

mHealth: mobile health
PRISMA-ScR: Preferred Reporting Items for Systematic Reviews and Meta-Analyses extension for Scoping Reviews
SEP: socioeconomic position
