# Supplementary material for: Investigating eHealth Lifestyle Interventions for Vulnerable Pregnant Women: Scoping Review of Facilitators and Barriers
Source: J Med Internet Res. 2024 Dec 20;26:e54366. doi: 10.2196/54366 (PMC11699491; doi:10.2196/54366)
Supplement: Multimedia Appendix 2 [file jmir_v26i1e54366_app2.docx]

**Multimedia appendix 2
Search strategy (original)**

| **Database searched** | **Platform** | **Years of coverage** | **Records** | **Records after duplicates removed** |
| --- | --- | --- | --- | --- |
| Medline ALL | Ovid | 1946 - Present | 1746 | 1718 |
| Embase | Embase.com | 1971 - Present | 2470 | 1072 |
| Web of Science Core Collection* | Web of Knowledge | 1975 - Present | 1865 | 488 |
| CINAHL** | EBSCO | 1982 - Present | 944 | 187 |
| Additional Search Engines: Google Scholar*** | | | 200 | 82 |
| **Total** | | | **7225** | **3547** |

*Science Citation Index Expanded (1975-present) ; Social Sciences Citation Index (1975-present) ; Arts & Humanities Citation Index (1975-present) ; Conference Proceedings Citation Index- Science (1990-present) ; Conference Proceedings Citation Index- Social Science & Humanities (1990-present) ; Emerging Sources Citation Index (2005-present)

**Limited to Academic Journals

***Google Scholar was searched via "Publish or Perish" to download the results in EndNote.

No other database limits were used than those specified in the search strategies

**medline 1746**

(Life Style/ OR exp Smoking/ OR exp Diet/ OR Eating/ OR exp Exercise/ OR exp Alcohol Drinking/ OR Stress, Psychological/ OR exp blood Pressure/ OR Body Weight/ OR exp Body Weight Changes/ OR Ideal Body Weight/ OR Body Mass Index/ OR exp Sleep/ OR Folic Acid/ OR exp Smoking Cessation/ OR Blood Pressure Determination/ OR Maternal Behavior/ OR Paternal Behavior/ OR (lifestyle* OR life-style* OR smoking* OR tobacco* OR diet* OR exercise* OR alcohol* OR stress* OR (blood ADJ3 pressure*) OR (body ADJ3 (weight OR mass)) OR (weight ADJ3 (change* OR gain* OR reduc* OR loss* OR loosing)) OR bmi OR sleep* OR overweight* OR obesit* OR folic-acid* OR (physical* ADJ3 (activ* OR inactiv*)) OR sedentary OR hypertens* OR ((parent* OR paternal* OR maternal* OR father* OR mother*) ADJ3 (behav* OR healthy))).ab,ti,kw.) AND (Online Systems / OR Telemedicine / OR Mobile Applications / OR Cell Phone / OR Internet-Based Intervention / OR Text Messaging / OR Social Media / OR online system/ OR (telehealth* OR ehealth* OR tele-health* OR e-health* OR mobile-health* OR (digital ADJ3 (health* OR intervention*)) OR m-health* OR mhealth* OR (mobile ADJ3 app*) OR smartphone* OR smart-phone* OR mobile-phone* OR cell*-phone* OR cellphone* OR web-base* OR webbase* OR internet-base* OR text-messag* OR sms OR social-media OR twitter OR instagram OR facebook OR telemedicine* OR telephone-base*).ab,ti,kw. OR (app OR apps).ti.) AND (exp Pregnancy/ OR Pregnant Women/ OR Prenatal Care/ OR Postpartum Period/ OR (pregnan* OR prenatal* OR pre-natal* OR perperium* OR perinatal* OR peri-natal*).ab,ti,kw.) AND english.la. NOT (exp animals/ NOT humans/)

**embase 2470**

(lifestyle/exp OR 'lifestyle modification'/de OR smoking/exp OR diet/exp OR 'dietary intake'/exp OR exercise/exp OR 'alcohol consumption'/de OR 'mental stress'/exp OR 'blood pressure'/exp OR 'body weight'/de OR 'body weight change'/exp OR 'body weight control'/exp OR 'ideal body weight'/exp OR 'lean body weight'/exp OR 'liveweight gain'/exp OR 'body mass'/de OR sleep/exp OR 'folic acid'/de OR 'smoking cessation'/de OR 'blood pressure monitoring'/de OR 'maternal hypertension'/de OR 'physical activity'/de OR 'parental behavior'/de OR 'maternal behavior'/de OR 'paternal behavior'/de OR (lifestyle* OR life-style* OR smoking* OR tobacco* OR diet* OR exercise* OR alcohol* OR stress* OR (blood NEAR/3 pressure*) OR (body NEAR/3 (weight OR mass)) OR (weight NEAR/3 (change* OR gain* OR reduc* OR loss* OR loosing)) OR bmi OR sleep* OR overweight* OR obesit* OR folic-acid* OR (physical* NEAR/3 (activ* OR inactiv*)) OR sedentary OR hypertens* OR ((parent* OR paternal* OR maternal* OR father* OR mother*) NEAR/3 (behav* OR healthy))):ab,ti,kw) AND (telehealth/de OR telemedicine/de OR 'mobile application'/exp OR 'mobile phone'/exp OR 'web-based intervention'/de OR 'text messaging'/de OR 'social media'/de OR 'online system'/de OR (telehealth* OR ehealth* OR tele-health* OR e-health* OR mobile-health* OR (digital NEAR/3 (health* OR intervention*)) OR m-health* OR mhealth* OR (mobile NEAR/3 app*) OR smartphone* OR smart-phone* OR mobile-phone* OR cell*-phone* OR cellphone* OR web-base* OR webbase* OR internet-base* OR text-messag* OR sms OR social-media OR twitter OR instagram OR facebook OR telemedicine* OR telephone-base*):Ab,ti,kw OR (app OR apps):ti) AND (pregnancy/exp OR 'pregnant woman'/de OR 'prenatal care'/de OR 'prenatal period'/de OR 'perinatal period'/de OR 'puerperium'/de OR (pregnan* OR prenatal* OR pre-natal* OR perperium* OR perinatal* OR peri-natal*):Ab,ti,kw) NOT ([conference abstract]/lim AND [2000-2019]/py) AND [english]/lim NOT ([animals]/lim NOT [humans]/lim)

**Web of science 1865**

TS=(((lifestyle* OR life-style* OR smoking* OR tobacco* OR diet* OR exercise* OR alcohol* OR stress* OR (blood NEAR/2 pressure*) OR (body NEAR/2 (weight OR mass)) OR (weight NEAR/2 (change* OR gain* OR reduc* OR loss* OR loosing)) OR bmi OR sleep* OR overweight* OR obesit* OR folic-acid* OR (physical* NEAR/2 (activ* OR inactiv*)) OR sedentary OR hypertens* OR ((parent* OR paternal* OR maternal* OR father* OR mother*) NEAR/2 (behav* OR healthy)))) AND ((telehealth* OR ehealth* OR tele-health* OR e-health* OR mobile-health* OR (digital NEAR/2 (health* OR intervention*)) OR m-health* OR mhealth* OR (mobile NEAR/2 app*) OR smartphone* OR smart-phone* OR mobile-phone* OR cell*-phone* OR cellphone* OR web-base* OR webbase* OR internet-base* OR text-messag* OR sms OR social-media OR twitter OR instagram OR facebook OR telemedicine* OR telephone-base*)) AND ((pregnan* OR prenatal* OR pre-natal* OR perperium* OR perinatal* OR peri-natal*))) NOT DT=(Meeting Abstract OR Meeting Summary) AND LA=(English)

**Cinahl 944**

(MH Life Style OR MH Life Style, Sedentary OR MH Life Style Changes OR MH Smoking+ OR MH Diet+ OR MH Eating OR MH Exercise+ OR MH Alcohol Drinking+ OR MH Stress, Psychological+ OR MH blood Pressure+ OR MH Body Weight OR MH Body Weight Changes+ OR MH Body Mass Index OR MH Sleep+ OR MH Folic Acid OR MH Smoking Cessation+ OR MH Blood Pressure Determination OR MH Maternal Behavior OR MH Paternal Behavior OR TI(lifestyle* OR life-style* OR smoking* OR tobacco* OR diet* OR exercise* OR alcohol* OR stress* OR (blood N2 pressure*) OR (body N2 (weight OR mass)) OR (weight N2 (change* OR gain* OR reduc* OR loss* OR loosing)) OR bmi OR sleep* OR overweight* OR obesit* OR folic-acid* OR (physical* N2 (activ* OR inactiv*)) OR sedentary OR hypertens* OR ((parent* OR paternal* OR maternal* OR father* OR mother*) N2 (behav* OR healthy))) OR AB(lifestyle* OR life-style* OR smoking* OR tobacco* OR diet* OR exercise* OR alcohol* OR stress* OR (blood N2 pressure*) OR (body N2 (weight OR mass)) OR (weight N2 (change* OR gain* OR reduc* OR loss* OR loosing)) OR bmi OR sleep* OR overweight* OR obesit* OR folic-acid* OR (physical* N2 (activ* OR inactiv*)) OR sedentary OR hypertens* OR ((parent* OR paternal* OR maternal* OR father* OR mother*) N2 (behav* OR healthy)))) AND (MH Online Systems OR MH Telemedicine OR MH Mobile Applications OR MH Cellular Phone OR MH Internet-Based Intervention OR MH Text Messaging OR MH Social Media OR TI(telehealth* OR ehealth* OR tele-health* OR e-health* OR mobile-health* OR (digital N2 (health* OR intervention*)) OR m-health* OR mhealth* OR (mobile N2 app*) OR smartphone* OR smart-phone* OR mobile-phone* OR cell*-phone* OR cellphone* OR web-base* OR webbase* OR internet-base* OR text-messag* OR sms OR social-media OR twitter OR instagram OR facebook OR telemedicine* OR telephone-base*) OR AB(telehealth* OR ehealth* OR tele-health* OR e-health* OR mobile-health* OR (digital N2 (health* OR intervention*)) OR m-health* OR mhealth* OR (mobile N2 app*) OR smartphone* OR smart-phone* OR mobile-phone* OR cell*-phone* OR cellphone* OR web-base* OR webbase* OR internet-base* OR text-messag* OR sms OR social-media OR twitter OR instagram OR facebook OR telemedicine* OR telephone-base*) OR TI(app OR apps)) AND (MH Pregnancy+ OR MH Pregnant Women OR MH Prenatal Care OR MH Postnatal Period OR TI(pregnan* OR prenatal* OR pre-natal* OR perperium* OR perinatal* OR peri-natal*) OR AB(pregnan* OR prenatal* OR pre-natal* OR perperium* OR perinatal* OR peri-natal*)) AND LA(english) NOT (MH animals+ NOT MH humans+)

**Google scholar**

lifestyle|"life style"|smoking|tobacco|diet|exercise|alcohol|stress|"body weight|mass"|"weight change|gain|reduction|loss"|bmi|"physical activity|inactivity" telehealth|ehealth|"e|mobile health"|"mobile app|application"|smartphone pregnant|pregnancy

**google**

lifestyle|"life style"|smoking|tobacco|diet|exercise|alcohol|stress|"body weight|mass"|"weight change|gain|reduction|loss"|bmi|"physical activity|inactivity" telehealth|ehealth|"e|mobile health"|"mobile app|application"|smartphone pregnant|pregnancy filetype:pdf -pmid -doi
